# Comparing quantum versus Markov random walk models of judgements measured by rating scales

**DOI:** 10.1098/rsta.2015.0098

**Published:** 2016-01-13

**Authors:** Z. Wang, J. R. Busemeyer

**Affiliations:** 1 School of Communication, The Ohio State University, Columbus, OH, USA; 2 Department of Psychology, Indiana University, Bloomington, IN, USA

**Keywords:** Markov models, quantum models, sequential effects, random walk models, first- and third-person judgements

## Abstract

Quantum and Markov random walk models are proposed for describing how people evaluate stimuli using rating scales. To empirically test these competing models, we conducted an experiment in which participants judged the effectiveness of public health service announcements from either their own personal perspective or from the perspective of another person. The order of the self versus other judgements was manipulated, which produced significant sequential effects. The quantum and Markov models were fitted to the data using the same number of parameters, and the model comparison strongly supported the quantum over the Markov model.

## 1. Introduction

One of the paradoxes explained by quantum mechanics is that the sequence of measurements affects the final observed results. Some physical variables, such as position and momentum, are non-commutative and classified as incompatible. The existence of in-compatibility led Niels Bohr to form his famous principle of complementarity. Sequential effects of measurement are not unique to physics. The order in which questions are asked has long been shown to influence human judgements in social and behavioural research [1]. Previously, we developed and tested a quantum probability model for question order effects obtained with binary valued (such as yes, no) questions [2], and we obtained surprisingly strong support for it using an *a priori* and parameter free test [3]. This study extends our previous work to multi-valued rating scales. We develop new quantum and Markov models for describing how people assign ratings to stimuli, and we empirically test and compare the two competing models by examining sequences of measurements.

## 2. Sequential effects and incompatibility

According to classical probability theory, events are represented as subsets of a sample space. If *A* and *B* are two events from the same sample space, then we can always define their conjunction by their intersection *A*∩*B*, and the latter is commutative so that *A*∩*B* = *B*∩*A*. Using these definitions, there should be no order effects because if we ask a question about *A* before *B*, then we should obtain *p*(*A*)⋅*p*(*B*|*A*)=*p*(*A*∩*B*), and if we ask the questions in the reverse order we should obtain *p*(*B*)⋅*p*(*A*|*B*)=*p*(*B*∩*A*). Therefore, we should find that *p*(*A*)⋅*p*(*B*|*A*)=*p*(*A*∩*B*)=*p*(*B*∩*A*)=*p*(*B*)⋅*p*(*A*|*B*).

To account for sequential effects with a classical probability model, we need to define the events not only with respect to the questions, but also in terms of the sequence. For example, using a Markov model, assuming we start the process in some state *S*, we can define the probability of the sequence *A* and then *B* as *p*(*A*_1_|*S*)⋅*p*(*B*_2_|*A*_1_). This will differ from the probability of the reversed sequence *p*(*B*_1_|*S*)⋅*p*(*A*_2_|*B*_1_). This provides one mathematical approach to modelling sequential effects of measurement.

Quantum theory accounts for sequential effects on measurements by using the concept of complementarity. When this concept is imported into psychology, it means that psychological measures, such as judgements, often require one to take different perspectives, which have to be taken sequentially, and the context generated by the first measure influences subsequent ones. It is an interesting twist of history that William James [4] introduced the concept of complementary mental ideas to psychologists before Bohr [5] introduced the concept of complementary physical measurements to quantum physicists.

According to quantum probability theory, events are represented as subspaces of a vector space. Each subspace is spanned by a subset of basis vectors. If *A* and *B* are two events that are spanned by the same basis, then they are compatible, and the sequence of questions does not matter. However, if *A* and *B* are two events that are spanned by different bases, then they are incompatible and the sequence does matter. Each subspace of a vector space corresponds to a projector. If two events are incompatible, then their projectors do not commute; otherwise, they are compatible. Therefore, in short, quantum theory uses the non-commutativity of projectors to account for sequential effects. Complementarity refers to the fact that some questions are incompatible.

Measurement sequence effects are typically regarded as ‘nuisance effects’ or ‘methodological artefacts’ by social and behavioural scientists [1]. However, from the quantum theoretical perspective, they are evidence for the essential constructive nature of human cognition [2]. Many cognitive measurements, such as attitude judgements and probability judgements, are incompatible measures, and are sensitive to the context created by previous questions. This is one of the important advantages to model human decision and cognition using quantum probability rules, and complementarity (or incompatibility) is an interesting and useful concept to be reintroduced into psychology.

An important issue for application of quantum theory to cognition is the following: Which questions are compatible and which ones are incompatible? This is largely an empirical question that can be answered by empirically examining sequential effects. However, one general idea is that questions are incompatible when the judge is required to undertake different perspectives or viewpoints in order to answer the questions. In other words, the person cannot judge or think about the two perspectives simultaneously. A prime example of perspective taking occurs when a person is asked to judge an issue from their own personal perspective as compared to judging the issue from someone else's perspective.

## 3. Review of self versus other judgements

First- and third-person effects are among the most robust phenomena in communication research. On the one hand, individuals tend to perceive that other people, particularly their anonymous peers, are more susceptible to the negative influences of media than they, themselves, are [6,7]. This effect has been examined in regards to many areas including pornography, television news, violent video games, and political and commercial advertising (see references below). On the other hand, when media effects are considered positive and thus desirable, individuals perceive themselves as more likely to reap the benefits of influence. This inverse phenomenon is known as the reverse third-person effect or first-person effect [8].

The fundamental process underlying first- and third-person effects has been attributed to social comparison [9]. The strength of the effect increases when the referent was described in increasingly socially distant terms [10]. The strength of the effect also increases as geographical ranges increased from participants’ hometown to the nation as a whole, suggesting that psychological distance and physical distance both impact the effect [11].

The process of switching from one's own perspective to another's requires a cognitive shift to imagine what the other person is thinking [12], which is costly in terms of cognitive resources [13]. Neuro-scientific research indicates that individuals’ brains mimic movement during this process, activating both spatial and emotional centres in the brain, allowing for cognitive travel into others’ perspectives in order to share experiences and empathize with them [14]. Interestingly, taking the other's perspective first may trigger a kind of identification that leads to this attenuating effect of the dissimilarity between the imagined other and the self.

To the best of our knowledge, only a few studies have explored the moderation on first- and third-person effects by the order of question, but the findings are mixed [15]. Some did not find any influence of the question order, while others identified one [16,17]. This moderation effect of order could potentially be the reason why studies have revealed mixed findings. A rigorous model is needed to formalize and understand these self–other judgements and how they depend on order.

Unlike physics, rules concerning compatibility of questions are not yet very well established, and psychologists are just beginning to explore these issues. Much more research in psychology is needed before we can firmly establish whether the compatibility of a pair of questions can vary across people, or even change with a person based on past experience with the pair of questions. With these cautions in mind, we selected self–other as a target for investigation because the findings reviewed above indicated that these questions are very likely to be incompatible for most people. In the current study, we asked judges to rate effectiveness of public service announcements (PSAs) from two perspectives. One was a general friends perspective which was compared with to the ‘self’ perspective. We expected these questions to be incompatible because people do not have much experience thinking about self and general friends simultaneously concerning issues about media messages. This lack of experience makes forming a conjoint perception about both self and friends unlikely, and instead, these judgements have to be made sequentially by changing perspectives from self to friends, or from friends to self, to answer the questions.

## 4. Methods

In total, 131 undergraduate students from a large Midwestern university participated in the study for course extra-credit. They were 19–30 years old (*M*=22.12, s.d.=1.42); 64.89% were female; and most were Caucasian (80.92%), followed by Asian (9.16%), African American (5.34%) and Hispanic (4.58%).

To ensure PSAs with a large range of emotional levels are included, a 3 (Valence: negative, positive, co-active) × 2 (Arousing Content: calm, arousing) × 2 (PSA repetitions) within-subjects design was employed. The experiment was conducted individually. On average, the experiment took around 30 min to complete. Each participant viewed and rated all the 12 PSAs. The order of the PSAs was randomized for each participant. After viewing each PSA, the participant was asked to judged the effectiveness of the PSA. They were asked ‘How effective is this message to you?’ and ‘How effective is this message to your friends?’ on a 9-point scale, where 1 means ‘not at all’ and 9 means ‘extremely’. The question order of the perceived effectiveness to self and to friends was randomized for each participant, which was logged by the computer software (self-then-friends, friends-then-self).

## 5. Empirical results

Below we present statistical tests to determine (i) whether or not there were differences between self versus other judgements and (ii) whether or not these judgements depend on order of questioning. Two methods were used to perform these tests. The first used statistical test of mean differences, and the second used *χ*^2^-tests of differences between the joint distributions of self by other ratings produced by each order.

### (a) Mean differences

The mean ratings for self and other, and for each question order, pooled across all PSAs and participants, are presented in table 1 (values shown in parentheses are predictions described later). Statistical tests of the mean differences were performed using Student's *t*-tests. First we computed the average rating for each person, averaged across all PSAs, and averaged across both orders, producing a separate average for self and for other judgements for each person. Then we computed the difference between the averages for self versus other for each person. The mean of these differences (*M*=0.37, s.e.=0.07) was statistically significant (*t*_130_=5.39,*p*<0.0001). Next we computed the average rating across all PSAs for each person, separately for self and other ratings when self was asked first, and also for self and other ratings when others came first. The mean difference between self versus other ratings was larger (*M*=0.50) when self was asked first as compared to when other was asked first (*M*=0.23). This interaction (*M*=0.50−0.23=0.27, s.e.=0.07) was statistically significant (*t*_130_=3.90, *p*=0.0002). The same conclusions were reached when using Wilcoxon signed-rank tests instead of Student's *t*-tests.

**Table 1.** Mean differences for self and other depending on order of question. Observed means are presented outside of parentheses; predictions from quantum model are presented inside parentheses.

*N*=131	self first	other first	avg.
self rating	4.64 (4.36)	4.11 (4.29)	4.38 (4.33)
other rating	4.14 (4.25)	3.88 (4.21)	4.01 (4.23)
avg.	4.39 (4.31)	4.00 (4.25)	4.20 (4.28)

### (b) Joint distributions

Tables 2 and 3 present the 9×9 joint distributions, separately for the self first question order and other first question order, respectively. The frequencies were computed by pooling across all 12 PSAs and pooling across all 131 participants, separately for each question order. The assignment of PSA to question order was randomized with equal probabilities, and this random sampling produced 775 observations in the self first order and 797 observations in the other first order (775+797=12×131). The rows labelled 1 through 9 represent the 9 rating levels for self-judgements, and the columns represent the 9 rating levels for other judgements, and each cell indicates the relative frequency (percentage) of a pair of judgements for one question order. The last row and column contain the marginal relative frequencies. The numbers in parentheses are predictions described later.

**Table 2.** Joint relative frequency distribution when self asked first. Observed outside parentheses, predicted inside parentheses.

*N*=775	1	2	3	4	5	6	7	8	9	sum
1	11 (12)	1 (1)	0 (0)	0 (0)	0 (0)	0 (0)	0 (0)	0 (0)	0 (0)	12 (13)
2	3 (4)	7 (8)	1 (1)	0 (1)	0 (0)	0 (0)	0 (0)	0 (0)	0 (0)	11 (14)
3	1 (0)	2 (4)	6 (7)	1 (1)	0 (1)	0 (0)	0 (0)	0 (0)	0 (0)	10 (13)
4	1 (0)	1 (1)	3 (3)	5 (6)	0 (1)	0 (0)	0 (0)	0 (0)	0 (0)	10 (11)
5	2 (0)	1 (0)	2 (0)	3 (3)	13 (12)	1 (1)	0 (1)	0 (0)	0 (0)	22 (17)
6	0 (0)	0 (0)	1 (0)	1 (0)	3 (2)	6 (6)	1 (1)	0 (1)	0 (0)	12 (10)
7	0 (0)	0 (0)	0 (0)	0 (0)	2 (0)	4 (2)	5 (4)	1 (1)	0 (1)	12 (8)
8	0 (0)	0 (0)	0 (0)	0 (0)	1 (0)	1 (0)	1 (3)	3 (2)	0 (1)	6 (6)
9	0 (0)	0 (0)	0 (0)	0 (0)	0 (0)	0 (0)	1 (0)	1 (3)	3 (5)	5 (8)
sum	18 (16)	12 (14)	13 (12)	10 (11)	19 (16)	12 (9)	8 (9)	5 (7)	3 (7)	100

**Table 3.** Joint relative frequency distribution when other asked first. Observed outside parentheses, predicted inside parentheses.

*N*=797	1	2	3	4	5	6	7	8	9	sum
1	16 (12)	3 (3)	1 (0)	0 (0)	1 (0)	0 (0)	0 (0)	0 (0)	0 (0)	21 (15)
2	2 (2)	9 (9)	1 (3)	1 (0)	0 (0)	0 (0)	0 (0)	0 (0)	0 (0)	13 (14)
3	1 (1)	2 (3)	7 (7)	2 (2)	1 (0)	0 (0)	0 (0)	0 (0)	0 (0)	13 (13)
4	0 (0)	1 (1)	2 (3)	6 (6)	1 (1)	0 (0)	0 (0)	0 (0)	0 (0)	10 (11)
5	1 (0)	1 (0)	1 (1)	2 (3)	11 (12)	1 (1)	0 (1)	0 (0)	0 (0)	17 (18)
6	0 (0)	0 (0)	0 (0)	1 (0)	3 (2)	5 (5)	0 (1)	0 (1)	0 (0)	9 (9)
7	0 (0)	0 (0)	0 (0)	0 (0)	1 (0)	2 (2)	6 (3)	0 (1)	0 (0)	9 (6)
8	0 (0)	0 (0)	0 (0)	0 (0)	0 (0)	0 (0)	2 (2)	2 (2)	0 (2)	4 (6)
9	0 (0)	0 (0)	0 (0)	0 (0)	1 (0)	0 (0)	0 (1)	1 (3)	2 (4)	4 (8)
sum	20 (15)	16 (16)	12 (14)	12 (11)	19 (15)	8 (8)	8 (8)	3 (7)	3 (6)	100

A *χ*^2^-test for order effects was computed using the joint distributions by comparing two models. The first model is the saturated model, which allows a joint probability for each cell and for each table. For the saturated model, each question order requires estimating 9×9 joint probabilities with the constraint that they all sum up to one, and so the saturated model entails a total of 9×9×2−2=160 parameters. The second model is the restricted model that assumes no order effects. This model assumes that there is a single joint distribution producing the results for both question orders, and so this model entails estimating only 9×9−1=80 parameters. We computed the log likelihood for each model and then computed the statistic *G*^2^=−2⋅[lnLike(saturated)-lnLike(restricted)]. The obtained value was *G*^2^=110.19. If we assume that the observations are statistically independent, so that this *G*^2^ statistic is approximately *χ*^2^-distributed, then the difference between models is significant (*p*=0.0143), and we reject the restricted model in favour of the saturated model. Rejection of the restricted model implies that question order made a significant difference in the joint distributions.

In summary, the empirical results demonstrate a robust difference between self versus other judgements. However, this difference depends on the question order with a larger difference produced when self-judgements are made first.

## 6. Quantum versus Markov models

Question order effects are intuitively explained by an ‘anchoring and adjustment’ procedure [9]: the answer to the first question provides an anchor that is then adjusted in light of the second question. However, these concepts have remained vague, and need to be formalized more rigorously. Both the quantum and Markov models provide more rigorous formulations of these intuitive ‘anchoring and adjustment’ type of processing ideas.

Before the PSA is presented, the judge is assumed to be in a state that is neutral with respect to each evaluation question. The PSA stimulus provides information for evaluating each question, and this evaluation process requires some period of time. The evolution of the evaluation is represented by a random walk-type process that starts from the initially neutral state, and drifts up or down the evaluation scale depending on the direction and strength of the PSA. Suppose a PSA is presented on a trial, and the person is asked a question about its effectiveness for self followed by a question about its effectiveness for other. To evaluate the first question about self, the person evolves from the initial state to a new state that reflects the effectiveness of the PSA from the perspective of self, which is used to select a rating for the question about self. After selecting the first rating for the self question, the state is revised to be consistent with this first answer, which provides the anchor for the anchoring–adjustment process. To evaluate the second question, the anchor provided by the previous state undergoes an adjustment process that evolves to another state reflecting the effectiveness of the PSA from the perspective of other. The state after the adjustment is then used to select a rating for the question about other.

Both the Markov and quantum models were based on the same measurement assumptions. This study used a 9-point rating scale to evaluate the PSAs. Although a 9-point rating scale is commonly used in social sciences, the number of scale values is somewhat arbitrary, and other scales can be used, such as a coarser 5-point scale, or a more refined 20-point scale. We assume that a person is capable of evaluating the stimuli on a fine internal scale comprising *N* evaluation states, ranging from state 1 (completely ineffective) and increasing by increments of one unit up to state *N* (completely effective). The first *n*_1_ evaluation states are assigned the first observed rating score equal to *R*=1, then next *n*_2_ states are assigned the next observed rating score equal to *R*=2, and so on. For a 9-point rating scale, the last *n*_9_ states are assigned the observed rating score equal to *R*=9. For both models, it was assumed that judges are capable of using a very fine lattice with approximately 100 states. More specifically, we set *n*_*k*_=11 evaluation states assigned to each rating scale value, and with *k*=1,9 rating scale values, this produces a total of *N*=9⋅11=99 evaluation states. We used an odd number for each category to allow for a midpoint within each category. We chose *N*=9⋅11=99 states because it approximates a continuum, and increasing the number *n*_*k*_ of states assigned to each rating produces practically the same results.

### (a) Markov random walk model

The Markov model was designed to be a version of the type of random walk decision process commonly used in other applications in cognitive science [18]. The Markov model is defined on a state space formed by a lattice comprising *N*=99 evaluation states {|*E*_1_〉,…|*E*_*i*_〉,…|*E*_*N*_〉} ordered according to effectiveness. To facilitate comparison with the quantum model, we can interpret these *N* states as *N* orthonormal basis vectors that span an *N*-dimensional vector space. A key property of the Markov model is that the same basis {|*E*_1_〉,…|*E*_*i*_〉,…|*E*_*N*_〉} is used to describe evaluations for self as well as for others. These *N* basis vectors can be numerically represented using the canonical basis, such that state |*E*_*i*_〉 corresponds to the *N*×1 vector *E*_*i*_=[0 ⋯ 1 ⋯ 0]^T^, where the number one is located in state |*E*_*i*_〉 and zeros elsewhere.

According to Markov theory, the person is located in exactly one state at any moment in time, but this location is not known to the theorist, and so we assign a probability that the person is in a state at each moment in time, to produce a mixture state. The mixed state of the Markov system at any point in time can be defined by a linear combination of the basis states

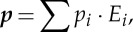

where *p*_*i*_ is a non-negative real number assigned to evaluation state |*E*_*i*_〉, and 
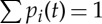
. The *N*×1 column vector ***p***=[*p*_*i*_] corresponds to the probability distribution across states at some point during the evaluation process. The basis vector *E*_*i*_ can be interpreted as a probability distribution over states for the special case in which we know the person is in state |*E*_*i*_〉 for sure.

The initial distribution at the beginning of a trial and before the PSA appears is defined as ***p***(0)=[*p*_*i*_(0)], with zeros assigned to all states except for 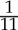 assigned to the 11 states |*E*_*i*_〉 with *i*=45,…,50,…55 in the neighbourhood of the neutral state |*E*_50_〉 corresponding to the middle rating *R*=5. This initial state is commonly used in other applications of Markov random walks in cognitive science [18].

Define ***T***=[*T*_*ij*_] as an *N*×*N* transition matrix, where *T*_*ij*_=*p*(|*E*_*i*_〉||*E*_*j*_〉) is the probability of transiting to state |*E*_*i*_〉 from state |*E*_*j*_〉. Then ***T***_S_ corresponds to evolution during the time period when evaluating the self question, and ***T***_O_ corresponds to evolution during the time period when evaluating the other question. Shortly, we describe how we construct these transition matrices, but first we complete the description of the anchoring and adjustment process used to compute the joint probabilities for each question order.

Define ***M***_*k*_ as a diagonal matrix that indicates the states corresponding to rating *R*=*k*. More specifically, ***M***_*k*_ is a diagonal matrix with zeros everywhere except for ones on the diagonal corresponding to the 11 rows (*k*−1)⋅11+1,…,*k*⋅11 which correspond to the rating *k*, for *k*=1,9. For convenience, define ***L***=[1 1 ⋯ 1] as a 1×*N* row vector of all ones that is used to sum a vector of probabilities across states.

If the self question is asked first, then the probability of a pair of ratings (*R*_S_=*j*,*R*_O_=*k*) for self and then other is
6.1
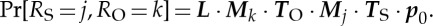

If the other question is asked first, then the probability of a pair of ratings (*R*_O_=*j*,*R*_S_=*k*) for other and then self is
6.2
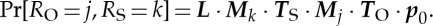



The transition matrices were constructed from a particular type of Markov random walk model called the continuous time parameter birth–death process with reflecting boundaries [19]. This is the same kind of Markov random walk model that has been used in other applications in cognitive science [18]. The transition matrices for Markov processes satisfy the Kolmogorov forward equation (d/d*t*)***T***(*t*)=***K***⋅***T***(*t*), which has the solution given by the matrix exponential 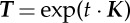, where ***K*** is the intensity matrix (or infinitesimal transition rate matrix). The intensity matrix ***K***=[*k*_*ij*_] is a tridiagonal matrix with entries *k*_*i*−1,*j*_=*α* in the upper diagonal and *k*_*i*+1,*j*_=*β* in the lower diagonal and *k*_*ii*_=−(*α*+*β*) on the diagonal. The difference 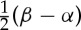 corresponds to what is called the mean drift rate of a random walk model, which determines the rate of movement in the increasing 
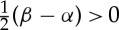
 or decreasing 
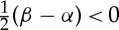
 direction along the lattice of states. The sum 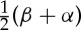 determines what is called the diffusion rate of a random walk process. We used an intensity matrix ***K***_S_ with intensity parameters (*α*_S_,*β*_S_) for the self-transition matrix, and we used an intensity matrix ***K***_O_ with intensity parameters (*α*_O_,*β*_O_) for the other transition matrix. The parameter *t* in the matrix exponential represents the time taken to process each message. However, this parameter can be absorbed into the intensity parameters. In sum, this model entails only four parameters to be estimated from the data.

### (b) Quantum walk model

The quantum model was designed to be similar to the type of dynamic and stochastic quantum processes that we have proposed in the past [20]. Like the Markov model, the quantum model uses a lattice of *N*=99 evaluation states ordered according to effectiveness. These *N* states are represented by *N* orthonormal basis vectors that span an *N*-dimensional vector space. Unlike the Markov model, however, the quantum model uses different bases for different questions. We use three bases: one is symbolized as {|*E*_1_〉,…|*E*_*i*_〉,…|*E*_*N*_〉}, which is a neutral basis that is used before any question is asked; the second is symbolized as {|*F*_1_〉,…|*F*_*i*_〉,…|*F*_*N*_〉}, which is used for questions about self; and the third is symbolized as {|*G*_1_〉,…|*G*_*i*_〉,…|*G*_*N*_〉}, which is used for questions about others. For convenience, we can numerically represent the neutral basis vectors {|*E*_1_〉,…|*E*_*i*_〉,…|*E*_*N*_〉} by the canonical basis, such that state |*E*_*i*_〉 corresponds to the *N*×1 vector *E*_*i*_=[0 ⋯ 1 ⋯ 0]^T^, where the number one is located in state |*E*_*i*_〉 and zeros elsewhere.

According to quantum theory, the person is not necessarily located at one basis vector, and instead the person's state is represented in the model as a superposition over several basis vectors at any moment. The superposed state of the quantum system at any point in time can be described by a linear combination of the neutral basis vectors

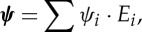

where *ψ*_*i*_ is a complex number assigned to evaluation state |*E*_*i*_〉, and 
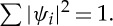
 The *N*×1 column vector ***ψ***=[*ψ*_*i*_] corresponds to the amplitude distribution across states at some point during the evaluation process. The basis vector *E*_*i*_ can be interpreted as an amplitude distribution over states for the special case in which the person would be observed in state |*E*_*i*_〉 for sure.

The initial distribution at the beginning of a trial and before the PSA appears is defined as ***ψ***(0)=[*ψ*_*i*_(0)], with zeros assigned to all states except for 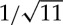 assigned to the 11 states *i*=45,…,50,…55 in the neighbourhood of the neutral state |*E*_50_〉 corresponding to the middle rating *R*=5. We chose this to make the assumptions for the quantum walk as similar as possible to the Markov random walk.

Define ***U***_S_ as an *N*×*N* unitary matrix, with element *u*_*ij*_=〈*F*_*i*_|*E*_*j*_〉 equal to the amplitude for transiting to the self state |*F*_*i*_〉 from the neutral state |*E*_*j*_〉. Define ***U***_O_ as an *N*×*N* unitary matrix, with element *u*_*ij*_=〈*G*_*i*_|*E*_*j*_〉 equal to the amplitude for transiting to the other state |*G*_*i*_〉 from the neutral state |*E*_*j*_〉. Then it follows that the product 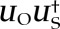 is the unitary matrix with element *u*_*ij*_=〈*G*_*i*_|*F*_*j*_〉 equal to the transition amplitude to state |*G*_*i*_〉 from state |*F*_*j*_〉, and 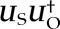 is the unitary matrix with element *u*_*ij*_=〈*F*_*i*_|*G*_*j*_〉 equal to the amplitude for transiting to state |*F*_*i*_〉 from state |*G*_*j*_〉. Shortly, we describe how we construct these unitary matrices, but first we complete the description of the anchoring and adjustment process used to compute the joint probabilities for each question order.

Similar to the Markov model, define ***M***_*i*_ as a diagonal matrix that indicates the states corresponding to rating *R*=*i*. More specifically, ***M***_*i*_ is a diagonal matrix with zeros everywhere except for ones on the diagonal corresponding to the 11 rows (*i*−1)⋅11+1,…,*i*⋅11 which correspond to the rating *R*=*i*, for *i*=1,9.

If the self question is asked first, then the probability of a pair of ratings (*R*_S_=*i*,*R*_O_=*j*) for self and then other is
6.3
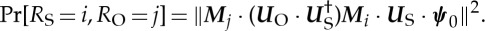

If the other question is asked first, then the probability of a pair of ratings (*R*_O_=*i*,*R*_S_=*j*) for other and then self is
6.4
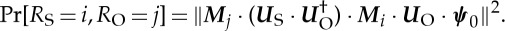



The unitary matrices were constructed from a particular type of quantum random walk model called the Feynman crystal model [21]. We have successfully used this model in other applications to cognitive science [22]. Unitary matrices for quantum models satisfy the Schrödinger equation (d/d*t*)***U***(*t*)=−*i*⋅***H***⋅***U***(*t*), which has the solution given by the matrix exponential 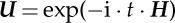, where ***H*** is the Hamiltonian matrix (a Hermitian matrix). The Hamiltonian matrix ***H***=[*h*_*ij*_] is a tridiagonal matrix. The entries *h*_*i*−1,*j*_=*α* above the diagonal and *h*_*i*+1,*j*_=*α* below the diagonal allow diffusion of amplitudes to adjacent states. The entries on the diagonal *h*_*ii*_=*β*⋅(*i*/*N*) serve as the potential function on the diagonal. The potential on the diagonal corresponds to a linear potential function that produces constant force in the direction determined by *β*. The off diagonal entries determine the diffusion rate. We used a Hamiltonian matrix ***H***_S_ with parameters (*α*_*S*_,*β*_S_) for the self unitary matrix, and we used a Hamiltonian matrix ***H***_O_ with parameters (*α*_O_,*β*_O_) for the other unitary matrix. The parameter *t* in the matrix exponential again represents the time taken to process each message. However, once again, this parameter can be absorbed into the Hamiltonian parameters. In sum, this model also entails only four parameters to be estimated from the data.

### (c) Theoretical differences between Markov and quantum models

Although there are many similarities between the Markov and quantum models, there are also some critical differences. One obvious difference is that the Markov process operates directly on probabilities, whereas the quantum process operates on amplitudes, and probabilities are based on the squared magnitudes of amplitudes.

Another important difference concerns the interpretation of the evaluation states. The Markov model relies on a single basis {|*E*_1_〉,…|*E*_*i*_〉,…|*E*_*N*_〉} for evaluating both self and other questions. However, the quantum model uses one basis {|*F*_1_〉,…|*F*_*i*_〉,…|*F*_*N*_〉} for self, and uses another basis {|*G*_1_〉,…|*G*_*i*_〉,…|*G*_*N*_〉} for other.

A consequence of the change in bases for the quantum model is the following. After answering the first question, the Markov and quantum models work differently. The Markov model transits directly from evaluation states consistent with the answer to the first question to evaluation states for answering the second question, using the same basis for both answers. The quantum model transits from evaluation states consistent with the first answer that are represented by the basis for the first question to evaluation states represented by the basis for the second question. To achieve the transition between different bases, the quantum model first transforms the amplitudes after the first question back to the neutral basis (e.g. applying the inverse operator *U*^†^_S_ when self is evaluated first), and then transforms this result into amplitudes for the basis for representing the second question (e.g. applying the operator *U*_O_ when other is evaluated second).

### (d) Non-judgemental processes

After analysing the results, we noticed that many participants had a tendency to skip over the judgement process on some trials and simply stick to the middle response of the scale at the rating *R*=5. To allow for this non-judgemental behaviour, we assumed that some proportion of trials were based on the random walk processes described above, and the remaining portion were based on simply selecting the rating *R*=5 for both questions. This was achieved by modifying the probabilities for pair of ratings by applying equations (6.1)–(6.4), with probability λ, and with probability 1−λ we simply set Pr[*R*_1_=5,*R*_2_=5]=1 and zero otherwise. When including this mixture parameter, both models entailed a total of 5 free parameters to be fitted from the data. Adding the mixture parameter only made modest improvements in both models, and all the conclusions that we reach are the same when this parameter was set equal to λ=1 (no mixture).

## 7. Model comparisons

Two different methods were used to quantitatively compare the fits of the quantum and Markov models to the two joint distributions produced by the two question orders.

The first method estimated the 5 parameters from each model that minimized the sum of squared errors (SSE) between the observed relative frequencies and the predicted probabilities for the two 9×9 tables. The SSE was converted into an *R*^2^=1−SSE/TSS, where TSS equals the total sum of squared deviations from both tables, when based on deviations around the mean estimated separately for each table. The parameters minimizing SSE for both the Markov and quantum models are shown in table 4. Using these parameters, the Markov produced a fit with a relatively low *R*^2^=0.54. It is important to note that the Markov can very accurately fit each table separately: *R*^2^=0.92 when fitted only to the self–other table, and likewise *R*^2^=0.92 when fitted only to the other–self table. However, different parameters are needed by the Markov model to fit each table, and the model fails when trying to fit both tables simultaneously. The quantum fits both tables simultaneously (using the same 5 parameters for both tables) with a much higher *R*^2^=0.90.

**Table 4.** Parameter estimates from Markov and quantum models. Note that the first four parameters include the effect of processing time for each message.

objective	model	*α*_S_	*β*_S_	*α*_O_	*β*_O_	λ	fit
SSE	Markov	339.53	330.37	419.82	402.93	0.90	*R*^2^=0.54
SSE	quantum	99.24	−14.57	89.53	−16.74	0.94	*R*^2^=0.90
*G*^2^	Markov	317.63	313.72	283.87	270.94	0.91	*G*^2^=1190
*G*^2^	quantum	114.58	−13.28	92.43	−17.56	0.91	*G*^2^=839

Using the parameters that minimize SSE, the joint probabilities predicted by the quantum model (multiplied by 100) for each table are shown inside the parentheses of tables 2 and 3. As can be seen, the predictions capture the negative skew of the marginal distributions as well as the positive correlation between self and other ratings. The means produced by these predictions are shown in the parentheses in table 4. As can be seen, the predicted means are close to the observed and ordered according to the observed means. The model correctly predicts self ratings to be higher than others, and that the difference is larger when self is rated first. However, the effects predicted by the model are smaller than the observed effects.

The second method estimated the 5 parameters from each model that maximized the log likelihood of observed frequencies from the two tables. The log likelihoods were converted into a *G*^2^ lack of fit statistic by comparing the 5 parameter models to the 180 parameter saturated model. The parameters minimizing *G*^2^ for both the Markov and quantum models are shown in table 1. Using these parameters, the Markov model produced a *G*^2^=1190, but the quantum model produced a lower discrepancy with *G*^2^=839. Both models use the same number of parameters and so a Bayesian information criterion would not change the conclusions. Although the quantum model fits the joint distributions better than the Markov model, both models produce deviations from the observed data. If we compare each 5 parameter model to the saturated model, and once again assume that the observations are statistically independent so that the *G*^2^ is *χ*^2^-distributed, then both models are statistically rejected when compared to the saturated model. This is not surprising given that both models are very simple and only use only 5 parameters to fit 182 observations.

In summary, both the Markov and quantum models were based on the same ‘anchoring and adjustment’ concepts, they both used walks driven up and down a scale of effectiveness by the PSA stimulus, they also used the same measurement model, and both were based on the same number (5) of parameters. The results of the comparison were the same when using both SSE and log likelihood methods—the quantum model produced substantially better fits than the Markov model.

## 8. Conclusion

This article makes two important contributions, one empirical and the other theoretical. Regarding the empirical contribution, we report evidence that if an individual is asked to make a pair of judgements about an issue from the perspective of self (what do I think?) versus another person's perspective (what does another person think?), then the pair of answers depends on the order that the question is asked. In particular, we found that ratings concerning the effectiveness of a public health service announcement are more pronounced for self as compared to others, but this effect primarily occurs when self is rated first.

These findings support our original hypothesis that self versus other judgements are incompatible in the quantum sense. That is, self versus other judgements require changing the basis used to represent the answers to questions from different perspectives. The incompatibility produced by changing between self versus other perspectives was predicted to produce the question order effects that we observed in this experiment.

Regarding the theoretical contribution, for the first time, we developed and quantitatively tested two different mathematical models for sequential effects obtained using multi-valued rating scales. One was a quantum walk model based on quantum probability principles, and the other was a Markov random walk model based on classical probability principles. Both models were developed from the basic idea that question order effects arise from a type of anchoring and adjustment process—the answer to the first question generates a context that influences the second question. Both models used the same number of parameters, which were used to fit to the two joint distributions produced by different question orders for self versus other judgements. The model fits clearly supported the quantum model over the Markov model.

An important question is why the quantum model fitted so much better than the Markov in this application. The two models were designed to be very similar—both used processes that produced evolution of evaluations across time, both used the answer to the first question to anchor the state before evaluating the second question, and both relied on the same measurement assumptions for mapping the evaluation states into the rating scale responses. Upon further analysis, we discovered that although the quantum model is capable of fitting both orders using the same 5 parameters, the Markov model can only fit the joint distributions accurately if we allow separate parameters for each question order. But the latter result leads one to question why the quantum model can fit both question orders using the same parameters but the Markov model cannot.

The main reason for the difference in model fits, we think, is that the quantum model changes the basis used to describe the state of the system that provides the evaluation of questions, whereas the Markov model relies on the same basis for describing the states when evaluating both questions. After answering the first question, the Markov model transits directly between states described by the same basis, whereas the quantum model transits between states represented by different bases. To achieve the transition between different bases, the quantum model first transforms back to the neutral basis, and then transits from the neutral basis to the basis for the second question. This return to the neutral basis before transiting to the basis for the second question may provide the main advantage for the quantum model. Perhaps the fit of the Markov model could be improved by introducing a partial return of the mixed state after the first question back to the initial state before applying the transformation for the second question. This decay back to the initial state would require adding an additional parameter to the Markov model to represent the amount of decay before adjusting for the second question. Note that a full return to the initial state would not work because that would produce no order effects at all.

Finally, we note that it is not necessary to directly pit Markov against quantum models as we have done here. Another theoretical approach to this empirical problem would be to construct a more general dynamical model that includes both Markov and quantum dynamics in a single master equation as suggested, for example, by Accardi *et al*. [23].
